# The Potential Impact of Directionality, Colour Perceptions and Cultural Associations on Disaster Messages During Heatwaves in the UK

**DOI:** 10.1371/currents.dis.775c310222d5829cb29b7a414370ca50

**Published:** 2015-04-14

**Authors:** Chris Tang, Gabriella Rundblad

**Affiliations:** Education and Professional Studies, King's College London, London, United Kingdom; Education and Professional Studies, King's College London, London, United Kingdom

## Abstract

The health risks posed by heatwaves have been well documented. In the UK, before and during a heatwave, alerts are issued to the general public based on a tiered warning system integrating the use of colour and number sequences. There has of yet been no formal assessment of the public response to these messages. Cultural and language barriers make some members of ethnic minority communities particularly hard to reach. These may be less challenging amongst younger community members, who may be well placed to instigate the circulation of warning information to those less able or willing to use conventional channels. This qualitative study assesses the role of age and ethnic and cultural background in the conceptualisation of the number and colour systems used as part of the Heat-Health Watch System (HHWS) and the National Severe Weather Warning Service (NSWWS). Young and older participants were recruited from the Bangladeshi and white British populations of Tower Hamlets. All participants were given a cognitive task that required them to identify and draw associations between 12 cards depicting the four colours and numbers used in the warning system and four pictures providing contextualisation in terms of heatwave risk. A qualitative analysis of the heuristics used in the group discussions provided insights into the conceptualisations basic to interpreting colour and number sequences as representations of risk graduations, and how interpretation might be influenced by age and ethnic and cultural background. There were considerable differences in the interpretation of young Bangladeshi and older white British participants, on the one hand, and older Bangladeshi participants, on the other. Young Bangladeshis and older white British participants conceptualised the colours and numbers as a vertical scale, with the numbers/colours at “the top” corresponding to representations of higher temperature. This conceptualisation was mainly based on strong associations between colour and temperature, with risk only associated with the upper limit of the scale. Older Bangladeshi participants, on the other hand, conceptualised the numbers and pictures as a narrative sequence and disassociated the colours from the other cards. The differences between groups suggest potential cultural barriers to the “intended” interpretation of the colour and number sequences for older Bangladeshis but not for young Bangladeshis. The fact that the predominant association for the colour sequence for both young Bangladeshis and older white British participants was with graduations of temperature rather than risk raises questions about the applicability of using colours in a tiered warning system.

## Introduction

Heatwaves have been linked with upsurges in mortality in Europe, Asia and the United States. Based on the excess of 14,947 deaths in France, between August 4 and 18, 2003, Poumadère and colleagues^1^ refer to heatwaves as a major mortal risk, number one among so-called natural hazards in post-industrial societies. The most vulnerable people during a heatwave typically belong to marginalised groups: the elderly, the infirm and the socially isolated. In the UK, it is the rapidly expanding and aging ethnic minority population^2^ that the current health plan aimed at reducing health risks has been unable to reach^3^ , largely due to language and cultural barriers^4^^,^ 5. Disaster communication about heatwaves in the UK relies on early warning systems that use meteorological information to predict periods of hot weather ahead of their occurrence. Kovats and Hajat^6^ describe the use of heatwave warning systems as a “success of public health”, yet, it is unclear how members of the public actually respond to warning information disseminated in the mass media prior to and during a heatwave.

While disaster communication with older ethnic minority groups can be hindered by language and cultural barriers^4^^,^ 5 , these may be less challenging amongst younger members of the community^7^ . This qualitative study reports on how younger and older participants from different cultural and ethnic backgrounds conceptualise the number and colour systems used as part of the Heat-Health Watch System (HHWS) and the National Severe Weather Warning Service (NSWWS) to communicate warning information during a heatwave. Our aim was to identify whether the age and ethnic and cultural background of the participants played a role in how they conceptualised key warning information. The implications for developing a disaster communication system that recruits young people to act as ambassadors of risk and health advice before or during a heatwave will then be discussed.

**The HHWS and NSWWS**

Public Health England and the Met Office operate an early warning system which comes into effect during the hot season. The HHWS integrates four warning levels[i]:

**Table:** Adapted from Public Health England 2014^3^

Level 1	Heatwave and Summer Preparedness
Level 2	Alert and Readiness when a heatwave has been forecast
Level 3	Heatwave Action when regional temperature thresholds have been reached
Level 4	Major Incident – Emergency Response

At each level, there are actions for health services, voluntary and community organisations and individuals. Efforts are currently underway to make this system consistent with the NSWWS, which is designed to prompt individuals to consider actions they may need to take in the case of severe weather. The NSWWS system involves the assignment of the colours referred to as “green”, “yellow”, “amber” and “red” to individual warnings:

**Figure:**
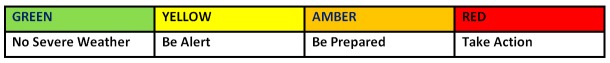
Adapted from http://www.metoffice.gov.uk/guide/weather/warnings

Either one or both systems tend to be referenced in mass media announcements, e.g. “yellow level 2 warning”. The combined system thus has four tiers, with “level one” alert status assigned the colour green, a “level two” alert the colour yellow, and so on, with a particular number (HHWS) or a particular colour (NSWWS) signalling a graduation of risk in association with impeding or manifesting hot weather.

**Taking into account an ethnically and culturally diverse audience**

A number of factors have been cited as barriers to disaster communication with ethnic minority communities, including lower educational levels and poorer literacy skills^8^, low levels of proficiency in the dominant language of the host culture^5^^,^ 9 and culturally rooted beliefs^10^. Some barriers, however, are only observable at a deeper, conceptual level. Both the HHWS and NSWWS require a degree of cultural knowledge to associate the colours and numbers with increasing levels of risk. The cultural association of particular colours with physical and abstract entities is well documented^11^^,^ 12. While there is limited evidence to suggest that particular colours placed in a particular order might be inherently perceived as indicative of increasing levels of risk^13^^,^ 14 , cross cultural differences in colour categorisation, naming and association^12^^,^ 15 ^,^ 16 make the existence of any universal pattern of correspondences unlikely. In one study, US and Chinese students rating colours on a scale of 1 to 9 yielded the following two sequences when ordered according to increasing hazardousness: blue, green, yellow, orange, red, and black for US students, compared with yellow, blue, green ,red, black and orange for Chinese students^17^^,^ ^33^.

In terms of representing risk as “lower” or “higher” using ordinal numbers, there is evidence to suggest a tier system may be more effective representation of different graduations of risk than a category system^18^. A conceptualisation of “more” as “up” (and “less” as “down”) may also be grounded in our universal, physical experience of the world: when a substance or a thing is added to a container or pile, the level goes up^19^ . On the other hand, speakers of different languages may have different spatial conceptualisations of abstract concepts, like risk. In a study of the metaphors used to represent stock fluctuations, verticality is a major factor for the use of spatial concepts in German texts, e.g. stocks “rise” and “fall”, while horizontality is dominant in Finnish texts, i.e. stocks “go forward” or “backwards”^20^. The writing direction of a particular language (top to bottom, right to left, or left to right) has also been found to influence space perception^21^^,^ ^23^, as well as how space is mapped onto conceptions of time^22^ and number^23^^,^ 24 .

**Accessing hard to reach communities through young people**

Potentially due to language and cultural barriers, a recurring observation in the rather sparse literature on how ethnic minority groups respond to warning information during disasters is their reliance on interpersonal networks over conventional channels^25^^,^ 26 ^,^ 27 ^,^ 28. Although strong interpersonal networks can strengthen a community’s resilience during a disaster, social networks may also end up perpetuating misleading beliefs and perceptions. Wolf and colleagues^29^, recruiting from communities in Norwich and London[ii], found that family members, friends and carers of older people in London felt that extreme heat neither posed a significant risk to them personally nor those they were supporting.

A number of global initiatives involving the recruitment of young people (children, adolescents and young adults) to act as ambassadors of risk and health advice before, during and after disasters have proven successful in accessing hard to reach communities^30^^,^ 31 ^,^ 32 ^,^ 33.The idea is that once educated about the risks, they will spread this knowledge in their immediate and broader social networks, prompting positive behaviour^32^. There is a particularly strong potential for these programmes in the current context. Young people are generally easier to reach and have a broader range of social opportunities for communicating health and warning information to the community^33^, and youth initiatives have been observed at their most effective when language barriers amongst their elders increase their agency in communities with a strong social cohesion but a high level of distrust in institutional sources of communication^34^, circumstances common to many ethnic minority communities^10^^,^ 35 ^,^ 36.

## Methods and Participants

In order to assess the potential for youth initiatives in the communication of warning information about heatwaves, this study sought to investigate how ethnic minority participants of different ages and cultural backgrounds conceptualised key aspects of the HHWS and NSWWS warning systems. This section overviews the study design, and the processes involved in analysing the data. A more detailed account can be found in Appendix 1.

The study draws on cognitive linguistic theory as its methodological basis. Cognitive linguistics sees language as embedded in both physiological and sociocultural realities, seeking to define both maximally schematic, universal properties as well as those that evolve through social interaction. This choice of methodology relates to the purpose of the study in uncovering both commonalities and differences in how members of different ages and ethnic backgrounds conceptualise warning information, and how this impacts on communication.

As studies of how ethnic minorities respond to warning information are few and far between, and the audience response to heatwave warning messages in the UK has yet to be documented, it was decided to take a qualitative approach to the investigation. As culture is inherently a group level phenomenon, we wished to observe conceptualisations as they emerged in peer interactions. Focus groups were therefore selected as the research method.

The London borough of Tower Hamlets was selected as a research site, as it combines a number of risk factors, including its urban location, high levels of socioeconomic deprivation, large ethnic minority population, lack of green space, high population density^37^ and high mortality rates for the over 65 year old age group^38^. Younger and older participants were recruited from the Bangladeshi population of Tower Hamlets, which is by far the largest ethnic minority group in the borough^39^. In addition, a control group of older participants were recruited from the white British population.

As significantly higher heat-related mortality is found amongst those over 65^40^^,^ 41 ^,^ 42, participants over 65 were sought for the older Bangladeshi and white British groups. The age threshold was lowered to 50 for the Bangladeshis due to the smaller size of the over 65 year old population in the borough. Additionally, people born in the UK aged between 16 and 24 with a Bangladeshi ethnic background were sought for the young Bangladeshi groups. Recruitment took place through local community organisations. Prior to participation, a screening tool was used to ensure participants matched the demographic criteria.

As we were advised that most older Bangladeshi participants had limited proficiency in the English language, two interpreters were recruited locally to moderate the focus groups. Both were fluent in English and Sylheti, the regional dialect of Bengali spoken in the Tower Hamlets community, and experienced in focus group moderation (see Appendix 1 for more details). Participants were offered a 20 pound incentive for their participation. Ethical approval was obtained from the E&M Research Ethics Panel at King’s College London (REP/13/14-122). Bangladeshi participants were divided into four groups segregated by age and (for culturally related reasons[iii]) gender. Two groups with older white British participants were also created. The process and purpose of obtaining informed consent and their data protection rights was explained on an information sheet. For older Bangladeshi participants, this information was mediated by the two moderators based on a translated, scripted version of the written materials.

As a component of the focus groups, participants were given a cognitive task, which generated the data reported in this paper. This task was designed to identify conceptualisations of the colour and numbers systems used in the HHWS and NSWWS. To identify potential communication barriers inherent in the use of colours and numbers as representations of risk graduations, the task was deliberately open ended. Participants were given 12 cards representing the four colours used in severe weather alerts (green, yellow, amber and red), the numbers referring to the four alert levels and, to provide contextualisation, four picture cards. In selecting the pictures, we were guided by depictions of temperature: Picture A depicts a grey, cloudy London skyline, Picture B depicts two people in a supermarket purchasing bottled water, Picture C shows a woman drinking water on a hot sunny day, and Picture D shows a man recovering from heat stress. For each of the three sets (colours, numbers and pictures) separately, participants were instructed to identify a logical order. The second part of the task required participants to draw associations between the ordered sets and thereby also reflect on their initial choices. This involved arranging the three sets of cards on a four by four grid. As there were more spaces on the grid than cards, participants were forced to decide on a horizontal or vertical representation. (Consequently, each final grid would contain an empty row or empty column). Photographs were taken of the completed grids for each focus group. While we might have engaged participants in an explicit discussion of the two warning systems, our primary objective was to identify the conceptualisations that underpin the interpretation of numbers and colours used in a tiered warning system, and how individual interpretations might vary based on different sociocultural experiences.

Prior to the focus group, participants were informed that they would be taking part in a small group discussion as part of a project seeking to improve health advice and warning information about the dangers of hot weather. To introduce the cognitive task, participants were told they were going to work together on a task that related to how we communicate about hot weather. They thus had limited information with which to contextualise what was represented on the cards and it was made clear throughout that there were no right or wrong answers. As we wished to see how groups from different age, ethnic and cultural backgrounds negotiated the task, the tasks were performed interactively, with limited moderator involvement. Participants were challenged to justify and explain their choices during and at the end of the activity. Although each group was encouraged to complete the task independently, in cases where individuals began to dominate, the moderator would intervene by ensuring all participants gave an opinion, and that the final choices that were made reflected, as far as possible, a group consensus.

All discussions were audio and video recorded, then transcribed. A translator experienced in Sylheti to English transcription was recruited to transcribe the older Bangladeshi discussions. As Sylheti has no written script, the Sylheti audio data was simultaneously transliterated. The translation process had two phases: a direct translation that captured the word order and literal sense of the original, and a glossed interpretation based on a systematic comparison of the transliterated Sylheti source and verbatim English versions. All data was fed into NVivo 10.

The photos of the completed grid arrangements were analysed, firstly, in terms of how the colours, pictures and numbers were sequenced and, secondly, in terms of whether participants had decided on a horizontal or vertical arrangement (see Appendix 1 for more details on coding). These findings were then correlated with the recorded data.

Codes were developed inductively from the transcribed and translated audio data to describe the heuristics and conceptual associations that guided the collaborations. The second phase of coding involved developing a coding tree based on the categorisation of the initial codes. Once the coding tree had been developed (see Appendix 1 for a description), it was possible to explore differences between groups by exploiting the coding functions available in NVivo 10. Coding tree maps were used to identify the dominant conceptualisations across and within groups (see Appendix 1). Analysis of the interactional data allowed dissenting opinions to be taken into account when identifying dominant patterns within and between groups. The video data enabled the identification of individual speakers and paralinguistic information that was essential to interpretation of the discussions, e.g. what participants were referring to with deictic references like “this” and “there”.

While, as a qualitative study, the data cannot represent the full heterogeneity of the various groups targeted, the sample size (see Table 1) falls within the ranges typically recommended for qualitative research^43^. Considering the experience of the research team (see Appendix 1), and the consistency of codes to emerge from the analysis of the audio data across groups, we considered the data sample to be sufficient for our purpose.

## Results

A total of 28 participants were recruited, with a relatively even distribution across the six focus groups (Table 1). Despite every effort to ensure an even gender distribution in the two older white British groups, one group was all female and the other contained three females and one male. Educational levels were well below average for all older participants. Most older Bangladeshi participants also had low levels of literacy and English proficiency (see Appendix 1 for more detailed information about participant characteristics).

**Table 1: Participants by group:** 

Group	Participants	Age range
A. Young Bangladeshi males	5	16-19
B. Young Bangladeshi females	6	16-24
C. Older Bangladeshi males	5	50 and older
D. Older Bangladeshi females	4	50 and older
E. Older white British, Group 1	4	65 and older
F. Older white British, Group 2	4	65 and older

The grid arrangements decided upon by the six groups are represented in Figure 1. In order to facilitate comparison between the arrangements, we have removed the empty row or column in each grid, and we consistently present the results for numbers first, then colours and last the pictures. The order of the individual sets of numbers, colours and pictures as well as whether participants opted for a vertical or horizontal representation have been preserved.

While none of the grid arrangements were identical, there were strong similarities in those completed by the young Bangladeshi and older white British groups on the one hand, and those completed by the two older Bangladeshi groups, on the other (Figure 1). Only the young Bangladeshi males decided on an arrangement that matched the HHWS and NSWWS systems in terms of number, colour and directionality. These three tendencies were largely supported by the analysis of the respective group discussions.

**Grid arrangements by all six groups:**
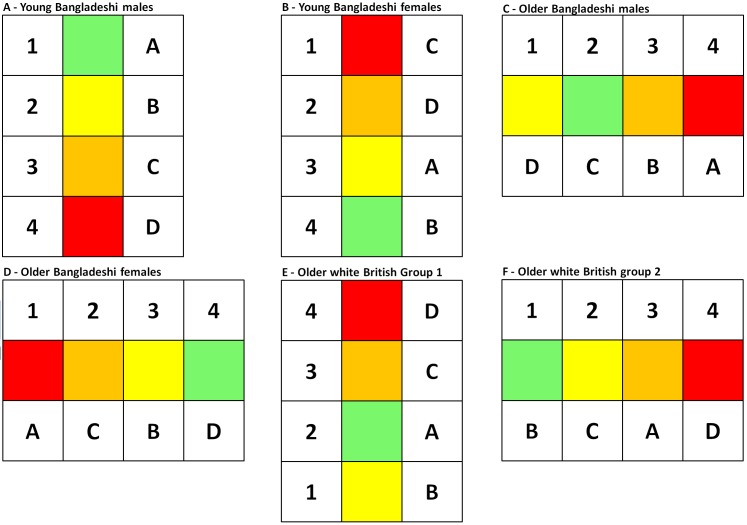

**Young Bangladeshi and older white British groups: colour and number as scales**

Young Bangladeshi and older white British groups (both groups 1 and 2) appeared to structure their arrangement of the colour and picture according to what upon what Johnson^44^ refers to as a “scale schema”. Scales are basic to understanding how numbers of objects, amounts of substances, degree or intensity can be more, less or the same, as well as our conceptualisation of number, amount, degree and intensity in terms of verticality, whereby “more” is perceived as “up” and “less” is perceived as “down”^44^. These characteristics of scalarity are well illustrated by the following discussion that took place while older white British participants were deciding on an order for the colour cards:

that- that’s but that green- green is like cool innit

yeah

green is a cool colour, innit

yeah

red’s a hot colour

red’s hot

orange can be

next one down

Older white British, Group 2

The association we observe here between colour and temperature (red with “hot” and green with “cool”) was predominant across all four groups. Red was invariability “hot”, with green and yellow typically designated as “cool” or “cold” colours. Participants in all four groups almost exclusively referred to the amber card as “orange”, and it rated in between red and the “cool” colours yellow and green. Red as the colour associated with the highest intensity of temperature is thus typically at the upper limit of the scale with the cooler colours lower down. In the young Bangladeshi male and older white British groups, there was some confusion over whether yellow should be rated as cooler (and therefore lower down) than green. This is reflected in the different orders for these two colours across the four groups (Figures 1A, B, E and F).

The four pictures were also associated with different intensifications in temperature, rather than graduations of risk. For instance, both groups of older white British and identified Picture B as the coldest, due an association between supermarkets and feeling cold. The associations of both colours and pictures with temperature meant that the two sets of cards could be ordered according to whether they were hotter or colder on a vertical scale of cumulative and rising intensity.

The upper limit of the scale was represented by the picture showing a man suffering from heatstroke (Picture D) in three of the four groups (young Bangladeshi males and both white British groups, but not young Bangladeshi females), and the colour red in all four groups (see Figures 1A, B, E and F). Picture D was consistently characterised in terms relating to excessive and/or dangerous levels of heat: “scorching”, “extreme” and “severe” (young Bangladeshi males); “danger”, “too hot”, “dangerous”, “sunstroke” (older white British, Group 1) and “too much sun” (Group 2). Although young Bangladeshi females did not, as the other groups, interpret Picture D as risk-related, they explained their choice of red at the upper extreme of the colour scale as it meant “like you’re burning like you’re to drop dead and really scorching hot like hell fire”. This negative characterisation apparent at the upper limit of the scale was never expressed in relation to the pictures or colours lower down, however. For young Bangladeshi males, for instance, the woman drinking water in Picture C “looks alright” compared with the “knackered man... ...sweating like mad” in Picture D.

In both young Bangladeshi groups, a scale schema also appeared to structure their conceptualisation of the colour sequence based on the physically distinctive (as opposed to associative) properties of individual colours. A male participant explained the colour sequence as a progression from “brightest to darkest”, and a female participant thought there was a movement from “lighter” colours to more “concentrated” ones, describing it as a shift in “the intensity of the colour”. It was not entirely clear whether participants were evaluating individual colours based on their position on the grey scale (colour value) or their proximity of on the colour spectrum (hue). Male participants, for instance, decided that yellow should follow green in the sequence due to its similarity with “orange”.

A scale schema was also the predominant heuristic in the arrangement of the number cards for young Bangladeshi male and older white British participants. In line with conceptualisation of “more” as “up”, an increase in number magnitude was associated with an increase in temperature on the scale, 4 representing the highest graduation and 1 the lowest:

...it’s one two three four it’s like steps on a highway, um how hot, the weather’s getting

Young Bangladeshi males

There were, however, inconsistencies between this conceptualisation and the physical arrangement of the cards on the grid. For young Bangladeshi males, the upper limit of the scale was actually at the bottom rather than the top of the grid (Figure 1A), while one group of older white British participants (Group 2) arranged the three sets of cards horizontally (see Figure 1F). When pressed about their choices, participants indicated the physical grid arrangement was incidental, the older white British participants maintaining the arrangement should be understood in terms of “up down” and not “side to side” regardless of how the cards were placed.

Contradictions were also apparent in the associations made by young Bangladeshi females between the numbers and the other two sets of cards. While the colour and picture cards were arranged according to their association with graduations of temperature, rising in intensity from the bottom of the grid to the top, the number cards were ordered so that the number with the lowest magnitude was at the top. A participant explained:

this is like a ranking, for how hot it is for number one being the hottest and the number four being, the coolest

Young Bangladeshi females

In this conceptualisation, it is the ordinal position of the numbers rather than magnitude that determines intensity, with the numbers at the start of the sequence associated with the hotter temperatures.

**Older Bangladeshi groups: Hot weather narratives**

Older Bangladeshi participants had difficulties engaging with all aspects of the task, relying heavily on moderator intervention and guidance. The picture cards prompted the most intensive discussions, with both sets of participants identifying common threads relating to hot weather and the consumption of water. Unlike the other groups, participants did not appear to draw upon a scale schema in deciding a logical order, focusing instead on sequential relationships between individual pictures. For Bangladeshi men, the woman drinking water (Picture C) should follow the man suffering from the heat (Picture D):

first the one where its hot, where he is tired, then one with drinking water

Older Bangladeshi males

Picture A, representing Parliament, is placed at the end as:

all these problems will be solved by the parliament

Older Bangladeshi males

The numbers, arranged horizontally running from left to right as a number line (Figures 1C and D), impose a narrative-like structure when associated with pictures. A female participant explains why Picture D follows Picture B:

for three [and] colour, people are bringing the water... ...so I gave that number three, and number four is not that much colour but he is nervous, he maybe drank water, maybe he will, but he has gone sick

Older Bangladeshi females

The juxtaposition of the number sequence (3, 4) onto the actions of obtaining (“people are bringing the water”) then consuming water (“he maybe drank, maybe he will”) gives the impression of a logical order that makes sense only as a temporal sequence. Although, for both groups, the narrative organisation is partial – the actors change and a single narrative structure does not hold for the entire sequence – the cards are arranged horizontally as would make sense for a narrative rather than a scale, with time thus following a path from left to right.

The colours, however, did appear to be conceptualised as a scale amongst female participants, with the moderator playing an interactive role in activating the schema. When the Bangladeshi women did not understand the initial instructions, the moderator prompts participants by asking:

which one is more and which one is less

Older Bangladeshi females

The women then proceeded to order the colours according to whether they were perceived as “more” or “less”, with “more” colours coming first in the final sequence. This heuristic appears to be based mainly on the direct physical properties of the colours, with “more” colours (red, orange) of a darker hue than “less” colours (yellow, green). It was hard to delineate any clear association between the colours and the other two sets of cards, however. There is a possible logic in the association of the “lightest” colour, green, and the heat sick man (Picture D). Both green, which is referred to as “not that much colour”, and paleness are cultural associations with lack of health. Yet, potentially due to the incongruity between a scalar conceptualisation and the heuristic used to order the other two sets of cards, no other correspondences are apparent for the other colours. Male participants also focused on physical colour properties, ordering colours according to hue: Green was associated with yellow; orange perceived as in between yellow and red. There was a similar disassociation of the colours from the other two sets, with no attempt made to find any correspondence.

## Discussion

The cognitive task was designed to assess how participants would conceptualise key aspects of the two warning systems, namely the numbers, colours and directionality (verticality and horizontality) in the context of heatwave risk. Of particular interest was whether the different cultural backgrounds and ages of the participants influenced their performance and the implications for the communication of warning information about heatwaves. The most striking differences occurred between the older Bangladeshi groups and other groups in all three aspects tested. The key factor underlying these differences is the extent to which conceptualisations of the three sequences were structured by a scale schema, with only limited activation of this schema evident from the older Bangladeshi discussions. In terms of directionality, scale schema activation tended to correspond with a vertical orientation, structured by the conceptual metaphor “more” is “up”, while older Bangladeshi participants identified narrative-like connections between the pictures, following a horizontal orientation.

Although the concept of scalarity is fundamental in explaining key differences, we should be wary of the assumption that older Bangladeshi participants did not draw extensively upon a scale schema because of fundamental differences in their conceptualisation system. Scales, at least in theory, are universally basic to our understanding of both physical and abstract entities, even when quantitative measurement is impossible^44^. The key distinction between groups is the extent to which participants drew on different properties of the colour and number sequences. Older Bangladeshi participants solely focused on the direct, physical properties of the colours and conceptualised the numbers as an ordinal sequence. In the other groups, we observed associations between colour and temperature, colour and risk and number magnitude and intensity. These associations draw upon cultural knowledge, specifically the practice of using numerical gradients and colours to calibrate intensity scales, and the categorisation of colours in terms of temperature (e.g. warm and cool) and colour/risk gradient correspondences.

As a scalar conceptualisation of colour and number sequences is fundamental to the interpretation of both tiered warning systems, younger Bangladeshi participants appear more likely than their older counterparts to draw on the cognitive resources required to interpret warning information about heatwaves in the UK, with young Bangladeshi males demonstrating the most flexibility. While a lack of cultural knowledge may have hindered the performance of the older Bangladeshi participants, it is difficult to determine without further investigation whether other factors, such as lower literacy levels and unfamiliarity with the task, may also have played a role. There are, however, direct parallels between task performance and level of interaction with the dominant culture. Studies of the Bangladeshi community in Tower Hamlets indicate the presence of a young, socially mobile group with bi-cultural identities alongside an older, more isolated one^45^. Amongst the younger group, social mobility appears to be more restricted when it comes to young women, with their changing social and cultural roles and expectations a source of intergenerational tension^45^. This suggests that young Bangladeshi males are the most likely to be able to draw on the cognitive resources necessary for accurate (i.e. intended) interpretation of warning information; which, in turn, suggests that they would potentially be more successful in its intercultural mediation in the community.

Although this was an open-ended task, the difficulties encountered by participants in associating numbers and colours raises a question mark over the use of colours to show graduations of risk. Three out of six groups (young Bangladeshi females and both older Bangladeshi groups) did not associate the colours with the number sequence in a way that suggested increasing magnitude, while the two white British groups ordered the colours differently than suggested in the NSWWS. A 1998 assessment of the colour coded flood alert warning system in the UK had similar findings^46^:

Colour coded warnings appear to be misunderstood by nearly all who receive them. This is because the colours are spontaneously linked with the escalating probability of flooding actually occurring, and not with the extant definitions to which the colours relate

The upper limit of a scale can offer an insight into its normative character, for instance, a lot of heat in the summer might be evaluated more negatively than in the winter^44^. While participants in the flood alert study misinterpreted the colour warnings as a probability scale, participants in the current study conflated risk and temperature. As a result, only the colour red, at the upper extreme of the scale, was consistently associated with both higher risk and higher temperature, with colours lower down associated with coolness rather than preparedness. This effect may have been enhanced as “colder” hues like green and yellow may appear colder when placed next to warmer hues and vice versa^47^.

On the other hand, the data suggests the use of a number-based tiered warning system may be an effective way of representing graduations of risk. Although there were some incongruities between the physical arrangements of the cards and the conceptualisation of “more” as “up”, this may have been related to the complexity of the task. For instance, even though young Bangladeshi males placed the number four at the bottom of their grid, it was still associated with the upper extreme of the scale[iv]. Similar incongruities were found in an experimental study where numbers were represented in a reverse orientation (i.e. right to left instead of left to right) in certain conditions, pointing to the pliability of the mental number line^48^. However, as demonstrated by young Bangladeshi females, successful interpretation relies on an association between number magnitude and intensity, which may not always be apparent, at least cross-culturally.

This study underlines the need, in risk communication systems, to account for the underlying conceptual processes involved in the interpretation of warning information, and how these can be influenced by environmental factors, particularly in a culturally diverse society. At the same time culture is far from being a stable entity. For instance, the recruitment though community organisations means that it is unlikely that the current participants represent the most isolated and hard to reach members of their respective communities. In addition, qualitative, potentially ethnographic, data are necessary to determine if the differences between groups observed in this study are evidence of differences in conceptualisation systems that can be identified at a cultural level.

Overall, these findings indicate that even if a more universally comprehensible communication system can be developed, significant communication barriers may still persist for older members of ethnic minority communities. As these barriers are cultural as well as linguistic, younger members benefiting from a greater degree of integration in the host culture appear to be well suited to the translation of key messages, at least in terms of the cognitive resources required to do so.

## Footnotes

[i]Not including the recent addition of Level 0, which refers to year round planning.

[ii]The ethnicity of participants was not specified.

[iii] Due to specifically defined gender roles in the Bangladeshi community, gatekeepers advised that gender segregation would be necessary to avoid situations where participants might feel uncomfortable participating or contributing to discussions.

[iv] This is in fact how the four levels of the HHWS are represented in the Heatwave Plan^3^.

## Appendix 1

### COREQ checklist

Based on the Consolidated Criteria for Reporting Qualitative Research (COREQ) checklist^49^.

**Table:**

**COREQ Category**	
**Domain 1: ** **Personal Characteristics**	
Moderator	The first Author moderated young Bangladeshi and older white British groups; the two members of the Swadinhata Trust moderated the older Bangladeshi groups.
Credentials	All three moderators hold an MA/MSc.
Occupation	The first author was a PhD candidate; the moderators of the female and male groups were working respectively as a freelance community development worker and a community researcher.
Gender	The older Bangladeshi female group had a female moderator, all other groups had a male moderator.
Experience and Training	At the time of the study, the first author had four years professional experience in linguistics and social science research, including focus group moderation and qualitative analysis; as well as qualitative research training at Masters and Doctoral level. The moderator of the female Bangladeshi group had actively facilitated/researched with individuals and groups and organisations since 1994. The moderator of the male group had ten years of experience of facilitating focus groups with Bengali speakers and conducting research in the local Bengali community.
Relationship established	The moderator of the female older Bangladeshi group had a prior informal relationship with one of the participants. There were no other prior relationships with participants in any of the groups.
Participant knowledge of moderator	Prior to the groups, participants were informed that they would be taking part in a small group discussion as part of a project seeking to improve health advice and warning information about the dangers of hot weather. The first author was introduced as the lead researcher in all groups. The function of the moderator in facilitating discussion was explained at the start of each group.
Moderator characteristics	The first author conducted the groups as part of an ESRC funded PhD study investigating the potential of young people to act as translators of health and warning information in relation to hot and cold weather. The moderators of the older Bangladeshi groups were recruited from an organisation seeking to promote culture, heritage and research relating to the Bengali communities in the UK.
**Domain 2: Study Design**	
Methodological orientation and theory	The study draws on cognitive linguistic theory as its methodological basis. Cognitive linguistics sees language as embedded in both physiological and sociocultural realities, seeking to define both maximally schematic, universal properties as well as those that evolve through social interaction. This choice of methodology relates to the purpose of the study in uncovering both commonalities and differences in how members of different ages and ethnic backgrounds conceptualise warning information, and how this impacts on communication.
Sampling	Participants were selected based on ethnicity, age and local residency. Prior to participation, a screening tool was used to ensure participants matched the demographic criteria.
Method of Approach	Community organisations that worked with target participants (youth groups, schools, community centres) were identified as gatekeepers. The gatekeepers identified and approached potential participants.
Sample size	28 participants took part in the five groups.
Non-participation	There were no drop outs for the groups with younger participants and older Bangladeshi participants. A total of 14 older white British participants reported interest when initially approached by the gatekeeper, but far fewer (eight) showed up on the day. This was reported to be normal for workshop attendance at the community centre.
Setting of data collection	To make sure participants felt comfortable contributing, all groups were held in familiar settings, e.g. their community centre or youth club.
Presence of non-participants	Only the moderator and participants were present for the older white British group. For the youth groups, the youth worker acting as gatekeeper was also present. The first author observed the two older Bangladeshi groups after being introduced to the participants.
Description of sample	Participant numbers, ages and ethnic backgrounds are provided in Table 1. Young Bangladeshi males and females were born in the UK, while older Bangladeshis had all moved to the UK as adults. Education levels were low amongst older participants from both ethnic backgrounds, with 70.6% (12/17) reporting they had no qualifications. Older Bangladeshi females also had no primary school education. In general, socioeconomic status appears to be well below average for London. 22.2% (6/27) reported they lived in a house they/or the parents partially owned, which is slightly below the average for the borough (26.7%), one of the most deprived in central and greater London. It is also worth noting that home ownership was mostly reported by young Bangladeshi female participants (four out of six cases).
Interview guide	The cognitive task was piloted prior to administration along with the other focus group materials.
Repeat interviews	The findings here will be carried forward in a second data collection looking at cold weather.
Audio/visual recording	All focus groups were both video and audio recorded.
Field notes	Field notes were made following initial recruitment discussions with gatekeepers and directly following each focus group. For the older Bangladeshi groups, the observer and moderators debriefed directly after each group.
Duration	The discussions prompted by the cognitive task produced between 10 and 15 minutes of audio/video data for each group. Each focus group yielded between approximately 1 hour and 1 and a half hours of recorded data.
Data saturation	While, as a qualitative study, the data cannot represent the full heterogeneity of the various groups targeted, the sample size (see Table 1) falls within the ranges typically recommended for qualitative research^43^ . Considering the experience of the research team (see above), and the consistency of codes to emerge from the analysis of the audio data across groups, we considered the data sample to be sufficient for our purpose.
Transcripts returned	As the participants were from groups typically defined as hard to reach, returning transcripts for comment would have been logistically challenging, particularly for illiterate participants.
**Domain 3: Analysis and Findings**	
Number of data coders	The visual data for the cognitive task was coded independently by the first and second authors. The video/audio data for the young Bangladeshi and white British groups was transcribed and coded by the first author, with codes checked, discussed and confirmed by the second author. For the older Bangladeshi groups, the data was first transcribed and translated by a trained linguist, fluent in English and Bengali (both Sylheti and standard Bengali), then coded by the first author. Codes were initially checked and confirmed with the translator and then checked, discussed and confirmed with the second author.
Description of the coding tree	The codes for the heuristics and conceptual associations derived from the data for the cognitive task divided into two central categories: Conceptualisations that appeared to demonstrate the activation of a scale schema and those that did not. Under the former category, we find evidence for a vertical conceptualisation of the colours/pictures/numbers, the various scales that emerged from the interactions (i.e. temperature, risk and colour) and conceptual associations that facilitate scale schema activation, e.g. the association of colour and temperature. In the latter category, we find categories relating to the narrative conceptualisation of the pictures, the conceptualisation of the number sequence as a number line and conceptual associations that did not relate to scalar conceptualisation of the colours/pictures/numbers.
Derivation of the themes	Codes were developed inductively from the transcribed and translated data to describe the heuristics and conceptual associations that guided the collaborations. The second phase of coding involved developing a coding tree based on the categorisation of the initial codes. Once the coding tree had been developed, it was possible to explore differences between groups.
Software	All data was fed into NVivo 10, which provides a range of coding functions. These were used to develop and explore coding categories. Coding tree maps were used to identify the dominant conceptualisations within and across groups by exploring references both quantitatively and qualitatively. For instance, the tree map represented in Appendix 2 is for the tree category coded* Scales*. The size of each of the subcategories *Temperature*, *Colour *and* Risk *is directly proportional to the number of references it comprises. Based on the number of references alone, *Temperature *clearly appears to be the most dominant category. Clicking on individual categories reveals all the references from the transcribed data, allowing a qualitative assessment of each reference, e.g. in terms of whether it represents an intense interaction between a number of participants or an isolated reference by a single participant.**
Participant checking	As the participants were from groups typically defined as hard to reach, returning transcripts for feedback would have been logistically challenging, particularly for illiterate participants.
